# Artificial Intelligence for Diagnostic and Prognostic Support in Breast Cancer: A Literature Overview

**DOI:** 10.3390/cancers18162723

**Published:** 2026-08-21

**Authors:** Diana Gina Poalelungi, Anca Iulia Neagu, Ana Fulga, Octavian Stefan Patrascanu, Iuliu Fulga

**Affiliations:** 1 Medical and Pharmaceutical Research Center, Faculty of Medicine and Pharmacy, “Dunărea de Jos” University of Galati, 800008 Galati, Romania; diana.poalelungi@ugal.ro (D.G.P.); octav974@gmail.com (O.S.P.); iuliu.fulga@ugal.ro (I.F.); 2 Saint John Clinical Emergency Hospital for Children, 800487 Galati, Romania; 3 Saint Apostle Andrew Emergency County Clinical Hospital, 177 Brailei St., 800578 Galati, Romania; 4 University Emergency Hospital of Bucharest, 169 Splaiul Independentei St., 050098 Bucharest, Romania

**Keywords:** artificial intelligence, digital pathology, breast lesions, breast cancer

## Abstract

Artificial intelligence (AI) is increasingly being integrated into medical practice, offering promising tools to improve diagnostic accuracy and clinical efficiency. In the field of breast pathology, AI applications, particularly those based on deep learning (DL) and machine learning (ML), are emerging as decision-support tools in both diagnostic and prognostic workflows. This review provides a comprehensive overview of current AI-based approaches, with a focus on their clinical utility in tumor detection, histological classification, biomarker assessment, and prediction of treatment response. In addition to summarizing available AI platforms, the review critically examines their level of clinical validation, regulatory status, and integration into routine practice. Key challenges are also discussed. Overall, AI is expected to play an increasingly important role in supporting pathologists and advancing precision medicine in breast cancer management.

## Simple Summary

This review provides a structured and clinically oriented analysis of artificial intelligence (AI) applications in breast pathology, highlighting their impact on diagnostic accuracy and prognostic assessment across key tasks, including morphological assessment of H&E slides, biomarker quantification using immunohistochemistry (IHC), lymph node metastasis detection, and risk stratification. By integrating evidence from currently available AI platforms, the review demonstrates variability in clinical maturity, regulatory status, and practical implementation, reflecting different stages of technological development and clinical integration.

## 1. Introduction

Breast cancer is the most common malignancy among women worldwide and represents a growing public health concern with significant socioeconomic impact [1], accounting for more than 2.2 million new cases in 2020 [2]. This burden is expected to increase further, with projections indicating that the global incidence of female breast cancer will reach approximately 3.2 million new cases annually by 2050 [3]. Despite advances in early detection and treatment, breast cancer remains a complex disease whose tumorigenesis is driven by a multifactorial interplay among genetic predisposition, lifestyle factors, and environmental exposures [4]. A better understanding of these risk factors by healthcare professionals is essential for developing targeted therapeutic strategies.

Histopathological examination remains the gold standard for diagnosing breast cancer, providing information on tumor cell morphology [5], mitotic activity [6], lymph node metastasis [7], and the host immune response, as reflected by the presence and density of tumor-infiltrating lymphocytes (TILs) [8]. Immunohistochemistry (IHC) enables pathologists to assess specific biomarkers, such as estrogen and progesterone receptors (ER/PR), human epidermal growth factor receptor 2 (HER2) expression, and the Ki-67 proliferation index, all of which are important for guiding treatment decisions and prognosis [9]. These assessments are time-consuming and may delay the diagnostic process, especially in high-volume clinical settings [10].

In recent years, whole-slide imaging (WSI) scanners have advanced substantially, enabling the generation of ultra-high-resolution digital images of conventional glass slides, thereby contributing to the development of digital pathology (DP) as a rapidly evolving field [11]. As a result, pathologists can perform digital primary sign-out, conduct remote consultations, and efficiently archive slides for teaching, research, and conferences [12]. More importantly, this digital framework enables the integration of AI algorithms into routine pathology, reducing workload, improving accuracy and efficiency, and providing new insights into disease prognosis and treatment response [13].

In breast pathology, several AI algorithms have been developed to support pathologists and reduce the time required for tasks such as tumor detection, breast cancer classification, grading, evaluation of lymph node metastasis, biomarker quantification, assessment of TILs, and prediction of BRCA1/2 mutation status [14].

This narrative review aims to provide a critical overview of recent advances in the application of AI to breast pathology, highlighting its applications in hematoxylin–eosin (H&E)-based morphological analysis, immunohistochemical biomarker quantification, and prognostic evaluation. Despite the rapid expansion of AI applications in breast pathology, structured comparisons integrating diagnostic roles, prognostic applications, and regulatory status into a unified clinical perspective remain limited [15,16]. To address this gap, the present review provides a clinically oriented classification of commercially available AI platforms according to their primary applications, summarizes their validation evidence and regulatory status, and discusses the challenges of integrating these technologies into routine breast pathology workflows.

The relevant literature was identified through targeted searches of PubMed, Scopus, Web of Science, and Google Scholar using combinations of keywords related to breast pathology, digital pathology, artificial intelligence, deep learning, machine learning, whole-slide imaging, breast cancer, histopathology, immunohistochemistry, HER2, ER, PR, Ki-67, lymph node metastasis, prognostic prediction, and digital pathology platforms.

The literature search included publications available through 31 July 2026, which was considered the final search date. No lower publication-date restriction was applied, although emphasis was placed on recent studies reflecting current developments in AI-based breast pathology.

Priority was given to original research articles, review articles, and regulatory or technical publications addressing the development, validation, clinical implementation, and performance of AI applications in breast pathology. Peer-reviewed validation studies were prioritized when assessing the performance and clinical validation of AI-based applications. Review articles were also used to provide broader context and to identify relevant primary studies. Publications were considered eligible if they addressed AI-based applications relevant to breast pathology, including diagnostic, prognostic, predictive, biomarker-assessment, or workflow-related applications. Publications unrelated to breast pathology, studies without a relevant AI component, duplicate publications, conference abstracts lacking sufficient methodological information, and sources for which adequate information could not be retrieved were excluded. Titles and abstracts were initially screened for relevance, followed by full-text assessment of potentially eligible publications. Reference lists of relevant publications were also screened to identify additional eligible studies. Because this review was designed as a narrative rather than a systematic review, study selection was guided by relevance to the predefined clinical and technological domains of the review, and no formal PRISMA-based selection process or quantitative evidence synthesis was performed. Details regarding the identification and evaluation of commercially available AI platforms are provided in Section 3.2.

The manuscript is organized as follows. The Introduction outlines the clinical background, rationale, and objectives of this review. Section 2 summarizes the histopathological diagnosis, classification, and prognostic assessment of breast cancer, providing the clinical background necessary for understanding AI applications in breast pathology. Section 3 introduces the principal AI methodologies used in computational pathology, reviews publicly available datasets, and presents a structured overview of commercially available AI platforms, classified according to their primary clinical applications and regulatory status. Section 4 critically discusses the current state of AI implementation in breast pathology, compares commercially available platforms with emerging research approaches, and addresses clinical validation, regulatory considerations, technical limitations, explainability, implementation challenges, and future research directions. Finally, Section 5 summarizes the main conclusions and clinical implications of this review.

## 2. Histopathological Diagnosis and Prognosis in Breast Pathology

### 2.1. Diagnosis

Breast carcinoma originates from the mammary epithelium and may develop through an in situ stage confined by the basement membrane, known as carcinoma in situ (CIS) [17]. Invasive carcinoma occurs when cancer cells acquire the ability to breach the basement membrane of the ducts and spread into the surrounding breast tissue [18]. This marks the transition from a localized, noninvasive stage to a more advanced and potentially aggressive form of the disease [19]. However, several benign conditions can closely resemble breast cancer, both clinically and radiologically, including fibroadenoma, fibrocystic changes, fat necrosis, sclerosing adenosis, and radial scars. These conditions may pose diagnostic challenges in the absence of histopathological confirmation [20]. In such cases, pathologists carefully examine the tissue architecture and cellular morphology under the microscope to distinguish between benign and malignant processes, often relying on additional IHC markers when necessary. Immunohistochemical markers such as p63, smooth muscle actin (SMA), and cytokeratin (CK) 5/6 help differentiate benign, in situ, and malignant breast lesions by highlighting myoepithelial cells, which are preserved in benign and in situ lesions but are lost in invasive carcinomas [21].

### 2.2. Classification

Breast cancer classification is commonly structured into histopathological, molecular, and biological subtypes [22]. Histopathological classification according to the 2019 World Health Organization (WHO) Classification of Tumors of the Breast is based on microscopic features and includes invasive carcinomas of no special type (NST), special types, and in situ lesions [23]. The classification also recognizes several rare tumor types, including neuroendocrine, fibroepithelial, mesenchymal, and hematolymphoid tumors [24].

Molecular classification is based on ERα, PR, and HER2 expression, together with gene expression profiling, and includes five intrinsic subtypes: luminal A, luminal B, HER2-enriched, basal-like, and normal-like [24]. These subtypes differ in their molecular characteristics, clinical behavior, prognosis, and response to systemic therapy [25,26]. Luminal A generally has the most favorable prognosis, whereas luminal B, HER2-enriched, and basal-like tumors are associated with more aggressive behavior and may require more intensive treatment strategies [27,28].

The biological classification categorizes breast cancers according to endocrine responsiveness as highly endocrine-responsive, non-endocrine-responsive, or of uncertain endocrine responsiveness, providing a framework for treatment selection [29]. Accordingly, endocrine therapy is recommended for highly endocrine-responsive tumors, whereas chemotherapy, with anti-HER2 therapy when appropriate, is recommended for non-endocrine-responsive tumors. In ER-positive/HER2-negative disease, combined treatment decisions are guided by established clinicopathological prognostic factors [30].

### 2.3. Prognosis

Major prognostic factors in breast cancer include histological subtype, Nottingham grade, tumor size, lymph node status, surgical margin status, presence of associated DCIS, lymphovascular invasion (LVI), expression of ER and PR, HER2 status, Ki-67 proliferation index, gene expression panels, response to neoadjuvant therapy, and TILs [31,32]. Histological subtype is determined by the tumor’s morphological growth pattern, with invasive carcinoma of no special type (NST) representing the most common subtype [33]. An overview of the principal histological subtypes according to the 5th Edition of the WHO Classification of Tumors of the Breast, including their general characteristics and prognostic relevance, is provided in Table 1.

The overview presented in Table 1 underscores the significant histopathological heterogeneity of breast carcinoma, encompassing a wide spectrum of entities that differ in architectural patterns, cellular morphology, and biological behavior [33]. While several subtypes, such as tubular, cribriform, and mucinous carcinomas, are associated with favorable outcomes, others, including metaplastic and neuroendocrine carcinomas, exhibit more aggressive clinical courses [87]. In addition, certain entities, such as invasive micropapillary carcinoma, are characterized by a high propensity for lymphatic spread, further emphasizing the prognostic variability across subtypes [88]. This heterogeneity highlights the complexity of histopathological diagnosis and prognostic assessment in breast pathology and represents a significant challenge for the development and implementation of AI-based diagnostic systems, which must be capable of accurately recognizing a broad range of morphological patterns, including rare and diagnostically challenging subtypes [89].

## 3. AI-Based Digital Pathology Platforms for Breast Cancer

Recent advances in AI are transforming breast cancer (BC) diagnostics by enhancing the accuracy, efficiency, and reproducibility of pathological assessment [90], while the growing availability of commercially deployed AI-based tools offers valuable decision support for pathologists and oncologists in clinical practice [91]. The increasing number of AI-related patents reflects sustained industrial investment in digital pathology and facilitates the rapid translation of research algorithms into commercial platforms. Consequently, numerous AI solutions have progressed from proof-of-concept research to commercially available systems supporting diagnostic, predictive, and prognostic applications in breast pathology [92].

**Figure 1:**
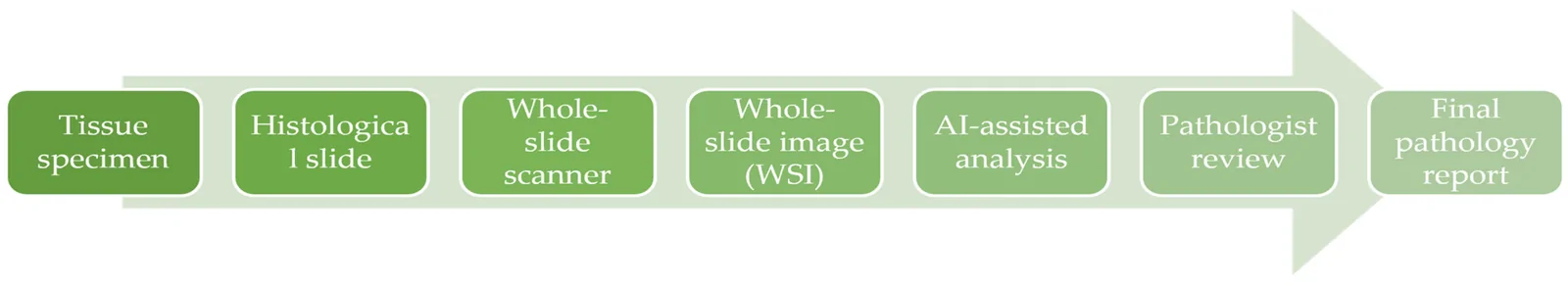
Overall workflow of AI-assisted digital pathology.

The overall workflow of AI-assisted digital pathology is illustrated in Figure 1.

### 3.1. AI Methodologies

AI methodologies applied in breast pathology encompass a broad range of approaches, including conventional machine learning methods, deep learning approaches, including convolutional neural networks (CNNs), transformer-based architectures, such as Vision Transformers (ViTs), foundation models, self-supervised learning approaches, multimodal AI systems, and emerging generative AI models [93].

Most contemporary AI platforms for breast pathology rely on DL, a subset of ML that automatically learns hierarchical image representations directly from raw pixel data [94,95]. In digital pathology (DP), convolutional neural networks (CNNs) remain the most widely adopted architecture due to their ability to identify increasingly complex morphological features through successive convolutional and pooling layers [96,97]. During training, these networks progressively learn representations ranging from low-level structures, such as nuclei and glandular contours, to higher-order tissue architecture, enabling automated tumor detection, tissue classification, grading, and biomarker assessment [93,97]. To further improve feature extraction and computational efficiency, modern commercial systems frequently incorporate advanced CNN architectures such as ResNet, DenseNet, and EfficientNet, which facilitate deeper network training, thereby improving diagnostic performance while reducing computational costs [98,99,100,101].

Although most AI applications for breast pathology are based on spatial-domain DL architectures, alternative analytical strategies have also been explored. These include frequency-domain and multiresolution approaches based on wavelet and wavelet packet transforms, handcrafted texture descriptors, and hybrid architectures combining wavelet-based feature extraction with CNNs, CNN–support vector machine (SVM) frameworks, or feature-fusion frameworks that combine handcrafted and DL features. By jointly exploiting spatial- and frequency-domain information, these approaches aim to improve multiscale feature extraction, texture characterization, and robustness to image variability. However, despite these encouraging results, they remain largely investigational and have not yet been incorporated into commercially available digital pathology platforms [102,103,104].

WSIs present additional computational challenges because their gigapixel resolution exceeds the processing capacity of conventional CNN-based workflows [105]. Consequently, most AI systems first divide WSIs into smaller image patches before analysis. Weakly supervised learning approaches, particularly multiple instance learning (MIL), have become widely adopted because they enable model training using slide-level labels without requiring exhaustive pixel-level annotations [106]. More recently, Vision Transformers (ViTs) have emerged as an alternative architecture that analyzes relationships between image patches using self-attention mechanisms, allowing more effective modeling of long-range spatial interactions across tissue sections [97,107].

### 3.2. Overview and Identification of Available AI Platforms

To provide a comprehensive overview of commercially available AI platforms for breast pathology, multiple sources were consulted, as no centralized registry is currently available. Commercial AI platforms were identified through targeted web searches using combinations of keywords such as “breast pathology AI”, “digital pathology AI”, “commercial AI pathology platform”, “HER2”, “ER”, “PR”, “Ki-67”, and “lymph node metastasis”, supplemented by the terms “software” and “algorithm”. Information on each platform was obtained from publicly available sources, including official company websites, product documentation, the published literature, and publicly available regulatory databases, from which relevant data on functionality, intended clinical use, and regulatory status were systematically extracted. Regulatory status (FDA clearance, CE-IVD marking, and research-use-only [RUO] designation) was verified using manufacturers’ official documentation and relevant regulatory resources in July 2026.

Peer-reviewed scientific evidence was distinguished from manufacturer- or company-provided information during data extraction. Claims regarding algorithm performance, diagnostic accuracy, and clinical validation were preferentially derived from peer-reviewed studies, with independent or external validation evidence highlighted where available. Manufacturer websites, product documentation, and regulatory databases were used primarily to verify product characteristics, intended use, commercial availability, and regulatory status, and were not considered equivalent to independent peer-reviewed validation evidence. For regulatory and product-specific information derived from manufacturer documentation or official company websites, the source type was explicitly identified in the corresponding references, together with the date of access. Regulatory information was preferentially cross-checked against publicly available regulatory sources, where available.

Currently available AI platforms for breast pathology can be broadly categorized by their primary clinical function, including morphological assessment of hematoxylin–eosin (H&E) slides, quantitative evaluation of immunohistochemical (IHC) biomarkers (such as HER2, ER, PR, and Ki-67), detection of lymph node metastases, and prognostic risk assessment and outcome prediction [15]. Representative examples include platforms developed by Visiopharm [108], Paige [109], Aiforia Technologies [110], Ibex Medical Analytics [111], Mindpeak [112], and Roche Digital Pathology [113], among others, each offering distinct capabilities and varying levels of clinical validation and regulatory authorization.

Depending on their intended use and geographic deployment, some of these systems are available as CE-IVD-marked or UKCA-certified solutions for clinical use in Europe [114], while others have obtained FDA clearance for use in the United States. A substantial number of AI tools, however, remain restricted to research use only, reflecting the ongoing evolution of regulatory frameworks and the need for further clinical validation [115].

Despite significant technological advances, most AI solutions remain task-specific and are typically deployed as decision-support tools rather than fully autonomous diagnostic systems, requiring integration into existing digital pathology workflows and continued expert pathologist oversight [116].

### 3.3. Publicly Available Datasets for AI Development in Breast Pathology

The development of AI models in breast pathology has been supported by several publicly available datasets that provide annotated histopathological images for algorithm training, benchmarking, and external validation. These datasets cover a wide range of diagnostic tasks, including tumor classification, lymph node metastasis detection, biomarker prediction, tumor proliferation assessment, and treatment response evaluation [117]. Table 2 summarizes the most widely used publicly available datasets for breast pathology AI research.

### 3.4. Functional Classification of AI Platforms

From a practical diagnostic perspective, AI tools in breast pathology can be organized based on their role within the histopathological workflow [15].

#### 3.4.1. AI for Morphological Assessment on H&E Slides

AI-based tools for morphological assessment of H&E slides focus on automated detection, classification, and grading of breast lesions, supporting pathologists in the initial stages of diagnosis [128]. Table 3 summarizes the main AI platforms available for H&E-based morphological analysis and their specific applications.

Table 3 demonstrates that most AI platforms developed for H&E-based analysis primarily focus on detection, classification, and grading tasks, reflecting their role in the early stages of the diagnostic workflow [137]. These tools typically rely on pattern recognition to identify suspicious regions, quantify histological features such as mitotic activity, and assist in tumor characterization [128]. Considerable variability exists among these platforms in terms of output, with many systems providing heatmap visualizations for region-of-interest prioritization rather than fully standardized diagnostic outputs [138]. This variability reflects the ongoing development of H&E-based AI applications, which remain less standardized than biomarker quantification tools.

#### 3.4.2. AI for Biomarker Quantification (IHC)

AI-based tools for biomarker quantification on IHC-stained slides enable automated detection and objective measurement of clinically relevant markers, supporting pathologists in therapeutic stratification and prognostic evaluation [139]. Table 4 summarizes the main AI platforms available for IHC-based biomarker analysis.

The analysis of available platforms highlights that most commercial platforms, including Aiforia Technologies [110], Mindpeak [112], PathAI [129], Tribun Health [132], Visiopharm [108], Ibex Medical Analytics [111], Roche [113], and Indica Labs [142], have developed breast cancer IHC suites capable of automated biomarker assessment, including detection and classification of tumor-cell nuclei as positive or negative [143]. In addition, several solutions provide advanced HER2 evaluation aligned with current guidelines, including the identification of HER2-low and HER2-ultralow categories [109]. Furthermore, Ki-67 quantification is performed through automated calculation of the proliferation index [108,110,111,113,129,132,134]. Compared with H&E-based applications, these tools demonstrate greater standardization and clinical maturity, reflecting the more structured nature of biomarker interpretation in routine practice. In contrast, a subset of platforms, such as DeepBio [130] and Primaa [131], remains under development for these applications, indicating that the field is still evolving. Finally, Paige [109] currently does not provide dedicated IHC biomarker quantification modules, reflecting its primary focus on H&E-based analysis.

#### 3.4.3. AI for Lymph Node Metastasis Detection

AI-based tools for lymph node assessment on H&E slides focus on the automated detection, localization, and quantification of metastatic deposits [144]. Commercial platforms enable automated detection of lymph node metastases, differing primarily in their output and sensitivity for small lesions [108,109,110,111,112,131]. Table 5 summarizes the commercial platforms and their output types.

The overview presented in Table 5 shows that AI-based tools for lymph node metastasis detection are primarily designed to support the identification, localization, and quantification of metastatic deposits on H&E-stained slides [120]. Most available platforms provide visual outputs, such as heatmaps or highlighted regions of interest. However, compared with IHC-based applications, AI-based lymph node metastasis detection remains less standardized. In addition, a significant proportion of tools are still under development or classified as RUO, particularly for the detection of small metastatic foci such as micrometastases and isolated tumor cells.

#### 3.4.4. AI for Prognostic Assessment and Risk Stratification

Certain AI platforms have been specifically developed for prognostic assessment and risk stratification rather than primary diagnostic tasks. Among these, Stratipath [146], Waiv [147], and PreciseDx [148] focus on extracting clinically relevant prognostic information from histopathological data. These tools typically generate risk scores, recurrence probabilities, treatment-response predictions, and tumor microenvironment (TME) characterizations, supporting personalized treatment decisions and patient stratification [149], as summarized in Table 6. Additionally, stromal biomarkers such as COL11A1 (collagen type XI alpha 1) have been associated with extracellular matrix remodeling, epithelial–mesenchymal transition, and therapy resistance, contributing to tumor progression and adverse prognosis in breast cancer [150]. Their prognostic value has been further supported by ML-based transcriptomic analyses with immunohistochemical validation. However, this evidence relates to molecular risk modeling rather than clinically implemented AI-based histopathology platforms [151].

Beyond dedicated histology-based risk prediction tools such as Stratipath Breast [146] and PreciseBreast [148], several other AI platforms contribute to prognostic assessment through complementary approaches. For instance, Lunit SCOPE IO [136] focuses on extracting quantitative features from H&E slides, including tumor–stroma ratio, immune cell infiltration, and spatial patterns, which can be incorporated into prognostic models. Similarly, Waiv [147] develops multimodal AI models that integrate histopathology with clinical and molecular data for outcome prediction.

### 3.5. Regulatory Classification of AI Platforms in Breast Pathology

AI platforms in breast pathology can also be categorized according to their regulatory status, which reflects their level of clinical validation, intended use, and readiness for routine diagnostic implementation [152]. Table 7 summarizes the main categories of AI platforms, distinguishing tools authorized for clinical use in the European Union (EU) and the United States (US) from those that remain investigational or research-oriented (RUO).

Table 7 highlights the heterogeneous regulatory landscape of AI platforms in breast pathology, reflecting different stages of clinical validation and implementation. Although several commercially available platforms have achieved CE-IVD or CE-IVDR certification for selected breast pathology applications [108,110,111,113,141], many AI tools described in the literature are still intended for research use only (RUO) or have not yet obtained regulatory approval. This disparity reflects the ongoing need for robust clinical validation, external evaluation, and regulatory assessment before widespread implementation in routine diagnostic workflows [112,113,129,133,134,136,140]. The regulatory designations reported in Table 7 refer to the specific AI modules and intended uses listed in the table and should not be interpreted as applying to the entire platform portfolio unless explicitly stated otherwise.

Notably, regulatory approval is more common for applications involving IHC biomarker quantification, where standardized scoring systems facilitate validation and clinical integration. In contrast, H&E-based algorithms and lymph node metastasis detection tools are more often limited to RUO status, reflecting the greater complexity and variability of these tasks.

## 4. Discussion

### 4.1. Current State of AI in Breast Pathology

The integration of AI into breast pathology is rapidly advancing, with an increasing number of platforms supporting different stages of the diagnostic and prognostic workflow [15]. As highlighted in this review, most current AI solutions for breast cancer assessment remain task-specific, focusing on distinct areas such as H&E-based morphological assessment, IHC biomarker quantification, lymph node metastasis detection, and prognostic evaluation. In real-world settings, the implementation of AI is often limited not by algorithm performance but by institutional constraints, costs, and resistance to changes in established pathology workflows [153].

Based on our comparative analysis of currently available platforms, one of the key observations is the clear functional separation among these tools. H&E-based platforms are predominantly designed for detection, classification, and grading and generally serve as decision-support systems in the early stages of the diagnostic workflow. In contrast, IHC-based applications appear to be more mature and standardized, enabling automated and reproducible quantification of key biomarkers such as ER, PR, HER2, and Ki-67, often in accordance with established clinical guidelines [137]. This difference likely reflects the inherently standardized and quantitative nature of biomarker assessment, which relies on well-defined scoring systems and relatively consistent visual patterns. Conversely, H&E evaluation involves more complex and heterogeneous morphological interpretation, which may explain the greater challenges associated with algorithm development, validation, and generalization in this setting [139].

Prognostic AI platforms represent a more recent area of development, aiming to extract complex information from histopathological images to predict patient outcomes, though many of these models require further validation. In a review of three studies evaluating AI-based prediction of gene mutations from H&E WSIs in breast cancer, Alam et al. [154] reported an average AUC of approximately 0.70. Among the evaluated molecular alterations, TP53 showed the highest predictive accuracy (AUC up to 0.78), whereas mutations such as PIK3CA, FOXA1, and MAP3K1 showed more modest performance. However, most published models were developed using The Cancer Genome Atlas (TCGA)-derived cohorts, and only one breast cancer study included external validation [155]. Therefore, despite promising preliminary performance, most mutation-prediction and prognostic AI models should currently be regarded as investigational and are not yet sufficiently validated for routine clinical use. Prospective validation in large, independent, multicenter cohorts is required to establish their generalizability, clinical utility, and robustness before implementation in routine practice.

An example of recent progress toward more robust validation is the genomics-driven AI model developed by Pareja et al. [156], which used biallelic CDH1 inactivation rather than histopathological diagnosis as the training ground truth. The model accurately classified invasive lobular carcinoma and achieved diagnostic accuracies of 0.95 in an internal validation cohort and 0.89 in an independent external cohort, illustrating how orthogonal genomic ground truth combined with external validation may improve the robustness and biological interpretability of AI systems. Even with further validation, the clinical utility of these models will ultimately depend on their integration with existing prognostic tools and treatment-decision frameworks; such integration remains limited in routine practice [157].

These differences illustrate distinct stages of development and clinical integration. IHC quantification is currently the most advanced and widely implemented area, with several CE-IVD-marked and FDA-cleared solutions available. H&E-based tools, although accurate, often remain in the early stages of clinical adoption. Prognostic models remain the least standardized, with challenges related to validation and clinical applicability.

AI-based tools for lymph node metastasis detection are an important component of breast cancer staging. However, this area remains less developed than H&E- and IHC-based applications. While several platforms can detect and localize metastatic deposits on H&E slides, their validation varies widely. Moreover, a substantial proportion of these tools are still under development or classified as RUO, reflecting the complexity of identifying small metastases such as micrometastases and isolated tumor cells. These limitations highlight the need for improved sensitivity and standardized reporting, particularly for clinically relevant small metastatic deposits.

From a regulatory perspective, the field remains heterogeneous. While some platforms have achieved CE-IVD marking, a substantial proportion remain classified as RUO. This highlights the need for more robust validation studies and more clearly defined regulatory frameworks to facilitate wider clinical implementation.

Regulatory authorization alone is not sufficient for safe clinical implementation. In addition to meeting regulatory requirements, AI systems should undergo continuous quality assurance, including local validation before deployment, routine performance monitoring, software version control, and periodic revalidation following major updates [158]. Furthermore, post-market surveillance and clinical governance frameworks are essential to monitor real-world performance, define institutional responsibilities, and ensure appropriate pathologist oversight and accountability throughout the AI-assisted diagnostic workflow [159].

It is important to note that AI is not intended to replace pathologists but rather to support and enhance their diagnostic accuracy and efficiency [15]. Successful integration into routine practice will depend not only on algorithm performance but also on workflow integration, time efficiency, and acceptance by clinicians, ultimately facilitating faster access to treatment for patients [160].

### 4.2. Current Limitations and Future Challenges

The application of AI in breast pathology has several important limitations. One of the most important challenges arises from preanalytical variability, such as poor tissue quality, staining artifacts, or inconsistent staining patterns, all of which can negatively affect the accuracy and reliability of automated diagnostic assessment [16]. In addition, significant variability exists across WSIs because of differences in file formats, scanner performance, and slide preparation [13]. To address these limitations, dedicated AI-based quality-control tools, such as SlideQC BF from Indica Labs [161], have been developed to automatically identify and flag suboptimal slides before analysis.

Beyond slide quality, AI performance is also influenced by interinstitutional variability resulting from differences in staining protocols, scanner technologies, and image acquisition workflows [162,163]. Even when tissue quality is adequate, variations in hematoxylin and eosin staining intensity, scanner optics, image resolution, color calibration, file formats, and compression algorithms may alter the visual appearance of histological structures, creating domain shifts between datasets [162,164]. Consequently, algorithms trained using slides generated by a limited number of laboratories or scanners may show reduced performance when applied to external cohorts. Several approaches, including stain normalization, color augmentation, and domain adaptation, have been proposed to improve model robustness [105,165]. A multicenter evaluation demonstrated that stain normalization can improve image consistency and reduce interlaboratory staining differences, although its effectiveness depends on tissue type and the normalization method employed [162]. More recently, Chai et al. demonstrated that staining and scanner variation can affect the performance and generalizability of pathology foundation models, with performance generally being highest when training and testing WSIs were acquired using the same scanner platform, while some models demonstrated greater robustness across scanner conditions [163]. These sources of variability collectively contribute to the phenomenon known as domain shift, in which the distribution of data encountered during clinical deployment differs from that used for model development. Consequently, AI algorithms should undergo external validation using independent datasets to establish their ability to generalize, followed by local validation to confirm consistent performance within the technical environment and workflow of the institution where they are implemented [164].

Validation of AI algorithms is required before their adoption for clinical use. Validation refers to the process of demonstrating that an algorithm performs accurately, reliably, and consistently across different datasets and clinical settings. Although several AI platforms have obtained regulatory authorization, such as CE-IVD marking or FDA clearance, this does not eliminate the need for further local validation to ensure reproducibility in real-world practice [166]. Validation evidence also differs substantially between commercially available platforms. Some solutions have been evaluated in multicenter studies involving independent cohorts and multiple scanners, whereas others are supported primarily by internal validation or technical performance reports. In addition, for some commercially available or RUO tools, publicly available evidence is predominantly manufacturer-provided, and independent peer-reviewed clinical validation remains limited or unavailable. Therefore, regulatory or commercial availability should not be interpreted as indicating an equivalent level of independent clinical validation across platforms.

For example, the Ibex Galen Breast algorithm underwent blinded external validation on 841 H&E breast biopsy slides from 436 patients across two institutions, achieving an AUC of 0.990 for invasive carcinoma detection and 0.980 for ductal carcinoma in situ (DCIS), followed by successful real-world deployment on 5954 routine biopsy cases while maintaining similarly high diagnostic performance [167]. Likewise, Cleo Breast was evaluated in a multicenter crossover clinical performance study involving 200 WSIs from four French institutions and 10 pathologists, demonstrating non-inferior diagnostic performance compared with conventional assessment, together with reductions in interpretation time of 9.6% for breast specimens and 32.4% for lymph node slides [168].

Validation evidence for AI-assisted IHC interpretation also varies considerably across commercially available platforms. The Mindpeak Breast ER/PR and Ki-67 solutions were validated in a routine diagnostic setting using 204 breast cancer slides processed across multiple laboratories, staining protocols, and scanner systems without laboratory-specific retraining, demonstrating non-inferior performance compared with conventional pathologist assessment [169]. Ibex Breast HER2 was evaluated in a multicenter, multireader study including 120 HER2 WSIs from four institutions; AI-assisted interpretation significantly improved interobserver agreement (75.0% vs. 83.7%) and overall scoring accuracy (85.3% vs. 88.0%), particularly for the clinically challenging distinction between HER2 0 and 1+ tumors [170]. Similarly, the Aiforia Breast Cancer Ki-67 solution underwent clinical validation and implementation at Mayo Clinic, where dedicated studies of accuracy, precision, interobserver agreement, intraobserver agreement, and multi-scanner reproducibility demonstrated excellent agreement with the reference standard (r^2^ up to 0.99), supporting its integration into routine pathology workflows [171]. In addition, HALO Breast AI (Indica Labs) has undergone multicenter validation for automated ER, PR, HER2, and Ki-67 assessment using datasets collected from three institutions and externally validated on independent cohorts, showing high concordance with expert pathologist scoring; however, these findings are currently available primarily as conference abstracts rather than full peer-reviewed publications [172].

Clinical validation of AI algorithms for lymph node metastasis detection has been reported for only a few commercially available solutions. Paige Breast Lymph Node was evaluated in a multireader study using 167 WSIs of sentinel lymph nodes interpreted by three pathologists with and without AI assistance. The algorithm significantly improved the sensitivity of metastasis detection, increasing it from 74.5% to 93.5% for two of the three participating pathologists, while simultaneously reducing the average review time by approximately 55% [173]. Similarly, the Visiopharm Metastasis Detection AI algorithm was validated in a routine digital pathology workflow using two sentinel lymph node cohorts (336 SLNs) and one non-sentinel lymph node cohort (258 LNs). In the sentinel lymph node validation cohort, the algorithm detected all 46 metastatic deposits, including macrometastases, micrometastases, and isolated tumor cells, achieving 100% sensitivity and a 100% negative predictive value. In the non-sentinel cohort, it likewise detected all 81 metastases with 100% sensitivity and a 100% negative predictive value, while significantly reducing pathologist review time [174].

Evidence supporting AI-based prognostic tools is emerging rapidly, although the level of clinical validation still varies among commercially available platforms. Stratipath Breast currently has the most extensive peer-reviewed clinical validation, having been externally validated in two independent multicenter cohorts comprising 2719 patients with ER-positive/HER2-negative breast cancer, demonstrating independent prognostic value for distant recurrence beyond established clinicopathological variables (HR = 2.76, 95% CI 1.92–3.97). Furthermore, the platform showed substantial concordance with the Prosigna genomic assay and successfully identified clinically relevant prognostic subgroups [175,176]. Similarly, the Waiv RlapsRisk BC platform has undergone large-scale external validation using seven retrospective cohorts comprising 6039 patients with HR-positive/HER2-negative breast cancer. The algorithm demonstrated robust prognostic performance across independent international cohorts and was further evaluated in the TAILORx population, where it provided prognostic information complementary to established molecular assays while maintaining consistent performance across different clinical settings [177].

Dataset bias represents another important limitation. Public datasets are often derived from a limited number of institutions, scanners, or staining protocols, which may cause AI models to learn technical or site-specific patterns rather than true biological signals [178,179,180]. Moreover, many publicly available datasets contain highly selected cases with relatively uniform slide preparation and imaging conditions, which may not adequately reflect the heterogeneity encountered in routine pathology practice [180]. Consequently, algorithms evaluated only on internal or publicly available datasets may demonstrate overly optimistic performance that cannot be reproduced in independent clinical cohorts [105,181]. Current recommendations therefore emphasize that external test datasets should be completely independent of the training cohort and include cases from multiple institutions, scanner vendors, staining protocols, and patient populations to provide a realistic assessment of model performance and identify potential hidden sources of bias before clinical implementation [182].

Another important limitation is the high cost of implementing digital pathology and AI systems. The adoption of these technologies requires investment in infrastructure, including whole-slide scanners, storage systems, and software platforms, which may represent a significant financial burden for many institutions [183]. Beyond the initial investment, ongoing costs related to software licensing, hardware maintenance, data storage, cybersecurity, system upgrades, and technical support further increase the total cost of implementation [157]. In low- and middle-income countries, these challenges are further amplified by limited healthcare budgets, insufficient digital infrastructure, and restricted access to advanced technologies [184]. In addition, there is often a shortage of trained personnel capable of implementing, maintaining, and validating AI systems, even when funding is available through governmental or international programs [159]. As a result, the integration of AI into routine pathology workflows remains uneven, and additional health economic studies are needed to determine whether these technologies provide sufficient clinical and economic benefit to justify widespread adoption [185,186].

A further key limitation is the explainability of AI models. Although advanced DL architectures, including CNNs and ViTs, can achieve excellent diagnostic performance, their decision-making processes often remain difficult for clinicians to interpret. This “black-box” behavior may reduce user confidence and hinder regulatory acceptance and routine clinical adoption. To address this limitation, explainable AI (XAI) techniques, including attention maps, saliency maps, and Gradient-weighted Class Activation Mapping (Grad-CAM), have been developed to highlight image regions that contribute most strongly to model predictions [185,186,187]. These visualization techniques can support pathologists by improving transparency, facilitating verification of AI outputs, and identifying potential prediction errors. However, current XAI methods provide approximations of model behavior rather than complete explanations and should therefore be regarded as complementary decision-support tools rather than definitive evidence of model reasoning [159].

Finally, data privacy and security represent important challenges. The development of AI models requires access to large volumes of sensitive data, including histopathological images, genomic information, and clinical records. Ensuring secure storage, transfer, and processing of such data is essential [188]. Moreover, the use of large-scale real-world datasets raises important concerns regarding informed consent, data governance, and compliance with privacy regulations. The implementation of robust cybersecurity measures and standardized data-sharing frameworks will therefore be critical for the safe and ethical integration of AI into routine breast pathology practice [93].

This review has several limitations. First, the literature search was restricted to major bibliographic databases and English-language publications, which may have resulted in the omission of relevant studies published elsewhere or in other languages. In addition, the included studies were highly heterogeneous with respect to datasets, AI methodologies, validation strategies, and reported performance metrics, limiting direct comparisons across studies. Finally, the rapid pace of AI development means that new algorithms, commercial platforms, and regulatory updates may have emerged after completion of the literature search.

Future developments in AI for breast pathology are expected to move beyond current task-specific, image-based approaches toward more comprehensive diagnostic frameworks integrating histopathological, molecular, radiological, and clinical information [12]. These integrated systems are expected to support increasingly complex clinical applications, including prediction of treatment response, estimation of recurrence risk, and identification of clinically relevant subgroups such as HER2-low and HER2-ultralow tumors or distinct immune microenvironments [189]. This evolution is likely to be driven by next-generation AI architectures, including ViTs, hybrid CNN–Transformer models, MIL approaches, and foundation models pretrained on large-scale pathology datasets, which provide improved contextual understanding, scalability, and reduced reliance on extensive manual annotations [93,106,107]. Future studies should therefore prioritize prospective multicenter validation, multimodal integration, federated learning to facilitate privacy-preserving collaboration, standardized benchmarking frameworks for objective comparison of AI systems, and the clinical implementation of these architectures to ensure robust, interpretable, and generalizable decision-support systems for routine breast pathology practice [16,93].

## 5. Conclusions

AI is rapidly reshaping the field of breast pathology, offering significant potential to improve diagnostic accuracy and efficiency. Current applications in breast pathology demonstrate clear benefits, particularly in biomarker quantification and selected diagnostic tasks, while more complex areas, such as lymph node metastasis detection and prognostic prediction, continue to evolve. Despite these advances, several challenges remain, including issues related to data variability, validation, costs, and implementation in routine clinical practice.

Overall, AI should be viewed as a powerful decision-support tool that complements, rather than replaces, the expertise of the pathologist. With continued technological development, robust validation, and improved integration into clinical workflows, AI has the potential to play an increasingly important role in personalized breast cancer diagnosis and management.

## Figures and Tables

**Table 1:** Overview of principal histological subtypes of breast carcinoma.

Histological Subtype	Characteristics and Prognosis
Ductal carcinoma in situ (DCIS)	DCIS is a noninvasive epithelial proliferation [34,35], with higher-grade lesions associated with an increased risk of invasive progression [36].
Lobular carcinoma in situ (LCIS)	LCIS is a noninvasive lobular proliferation associated with an increased risk of invasive breast cancer [37,38].
Invasive breast carcinoma, no special type (IBC-NST)	IBC-NST is the most common invasive breast carcinoma [39], with prognosis determined by established clinicopathological and predictive biomarkers [40,41,42,43,44].
Invasive lobular carcinoma (ILC)	ILC is typically low-grade and ER-positive, with E-cadherin alterations and an increased risk of late distant recurrence [45,46,47,48].
Tubular carcinoma	TC is a well-differentiated invasive carcinoma with a favorable prognosis [49,50].
Invasive cribriform carcinoma (ICC)	ICC is characterized by a cribriform growth pattern and an excellent prognosis [51,52].
Mucinous carcinoma	Mucinous carcinoma is characterized by abundant mucin production and generally has a favorable prognosis [53,54].
Microinvasive carcinoma	Microinvasive carcinoma is defined by an invasive focus ≤1 mm and generally has a favorable prognosis [55,56,57].
Invasive papillary carcinoma (IPC)	IPC is a rare invasive breast carcinoma with a generally favorable prognosis [58,59].
Invasive micropapillary carcinoma (IMPC)	IMPC is associated with frequent lymphatic invasion and lymph node metastasis [60].
Carcinoma with apocrine differentiation	Carcinoma with apocrine differentiation shows distinctive apocrine morphology and variable prognosis [61,62,63,64,65].
Metaplastic carcinoma	Metaplastic carcinoma is an aggressive, typically high-grade and triple-negative breast carcinoma with squamous and/or mesenchymal differentiation [66,67].
Mucinous cystadenocarcinoma	Mucinous cystadenocarcinoma is an extremely rare breast carcinoma, generally associated with favorable outcomes [68,69].
Acinic cell carcinoma (AcCC)	AcCC is a rare salivary gland-type breast carcinoma with a generally favorable prognosis [70,71].
Adenoid cystic carcinoma (AdDC)	AdDC is a rare salivary gland-type breast carcinoma with a generally favorable prognosis [72,73,74,75].
Secretory carcinoma (SBC)	SBC is a rare breast carcinoma characterized by the ETV6::NTRK3 fusion and generally favorable prognosis [76,77,78].
Mucoepidermoid carcinoma (MEC)	MEC is a rare breast carcinoma with favorable outcomes in low-grade and aggressive behavior in high-grade tumors [79,80,81].
Polymorphous adenocarcinoma (PAC)	PAC is a rare salivary gland-type breast tumor with a generally favorable prognosis [82].
Tall cell carcinoma with reversed polarity (TCCRP)	TCCRP is a rare breast carcinoma with indolent behavior and generally favorable prognosis [83,84].
Neuroendocrine carcinoma (NEC)	NEC is a rare, high-grade breast carcinoma with neuroendocrine differentiation and poor prognosis [85,86].

**Table 2:** Overview of publicly available datasets for AI development in breast pathology.

Dataset	Material	Data Type	Main Application	Key Characteristics	Reference
BACH	Breast histology	Slides and WSIs	Classification	400 images 4 diagnostic classes (normal, benign, in situ, invasive)	[118]
BreakHis	Breast histology	Slides	Classification	7909 images from 82 patients 4 magnifications	[119]
CAMELYON16	Sentinel lymph node	H&E WSIs	Metastasis detection	Pixel-level annotations, benchmark challenge	[120]
CAMELYON17	Sentinel lymph node	H&E WSIs	Metastasis detection	Multicenter dataset, TNM labels	[121]
TCGA-BRCA	Breast cancer	H&E WSIs + clinical/genomic	Prognosis and molecular prediction	3111 WSIs with molecular and clinical data	[122]
TUPAC16	Breast cancer	H&E WSIs	Tumor proliferation	Mitotic score and PAM50 proliferation score	[123]
BRACS	Breast lesions	WSIs + ROIs	Classification	Seven lesion categories including atypia	[124]
BreastPathQ	Breast cancer	WSIs/patches	Tumor cellularity	Response to neoadjuvant therapy	[125]
HER2-Warwick	Breast cancer	WSIs	HER2 scoring	Ground truth HER2 scores	[126]
HEROHE	Breast cancer	WSIs	HER2 prediction	Prediction of HER2 status from H&E	[127]

**Table 3:** AI platforms available for H&E-based analysis of breast cancer.

Platform	Task	Input	Output	Reference
Paige Breast Detect & Neoplasm	Tumor detection and classification	H&E breast biopsy/excision WSI	AI-generated heatmap highlighting suspicious cancer and precancerous regions	[109]
Paige Breast Mitosis	Mitotic figure detection and quantification	H&E-stained breast WSIs	Identification of individual mitotic figures, mitotic hotspot localization, automated mitotic count, and mitotic density	[109]
Paige HER2Complete	HER2 expression prediction	H&E-stained breast WSIs	AI-based prediction of HER2 expression, including identification of HER2-low tumors	[109]
Aiforia Breast Cancer Grading	Histological grading	H&E-stained breast WSIs	Nottingham histological grade (tubule formation, nuclear pleomorphism, mitotic count)	[110]
Ibex Breast H&E	Breast lesion detection, classification, and grading	H&E-stained breast WSIs	Detection and classification of breast lesions (e.g., IDC, ILC, DCIS), DCIS grading, identification of microinvasion, microcalcifications, LVI, TILs, and heatmap visualization	[111]
Mindpeak Breast H&E	Tissue segmentation and tumor detection	H&E-stained breast WSIs	Tissue segmentation and identification of invasive tumor regions	[112]
PathAI TumorDetect	Case prioritization, tumor detection, and tissue quantification	H&E-stained breast WSIs	Case classification, tumor detection, and quantification of tumor, stroma, and necrosis	[129]
DeepBio DeepDx Breast	Tumor detection and classification	H&E-stained breast WSIs	IDC/DCIS detection, ROI highlighting, and tumor-to-tissue ratio quantification	[130]
Primaa Cleo Breast	Tumor detection, mitosis detection, and tissue feature analysis	H&E-stained breast WSIs	Detection of invasive and in situ carcinoma, heatmap visualization, mitotic count with hotspot identification, calcification detection, and TIL/nuclear atypia assessment (under development)	[131]
Tribun Health Mitosis Breast	Mitotic figure detection and quantification	H&E-stained breast WSIs	Automated mitotic figure detection and quantification	[132]
Gestalt Mitotic Cell Counting	Mitosis detection and hotspot identification	H&E-stained breast WSIs	Detection/highlighting of mitotic figures and automated identification of the 10-HPF region with highest mitotic activity	[133]
Indica Labs HALO—Breast Macrodissection AI	Tumor content quantification and tissue/cell analysis	H&E-stained breast WSIs	Quantification of tumor content, detection of noninvasive epithelium and necrosis, artifact exclusion, cell phenotyping, density heatmap generation, and estimation of tumor and necrotic cell percentages	[134]
Owkin/Waiv BRCAura RUO	BRCA1/2 mutation status prediction	H&E-stained breast WSIs	AI-based prescreening and prediction of germline BRCA1/2 mutation status	[135]
Lunit SCOPE IO	Pan-cancer H&E image analysis	H&E-stained breast WSIs	Pan-cancer H&E analysis, including lymph node metastasis detection and tumor microenvironment/biomarker assessment	[136]

**Table 4:** AI platforms available for IHC-based analysis of breast cancer.

Platform	ER IHC WSIs	PR IHC WSIs	Ki-67 IHC WSIs	HER2 IHC WSIs	Reference
Aiforia Breast Cancer Suite	√	√	√	√	[110]
Mindpeak Breast	√	√	√	√	[112]
PathAI IHC Breast Panel	√	√	√	√	[129]
DeepBio Breast Resection	Under development				[140]
Primaa Cleo Breast	Under development				[131]
Tribun Health	√	√	√	√	[132]
Visiopharm	√	√	√	√	[108]
Ibex Breast	√	√	√	√	[111]
Roche Digital Pathology	√	√	√	√	[113]
Indica Labs HALO Breast IHC	√	√	√	√	[141]

Note: √ indicates that the corresponding feature is available.

**Table 5:** AI platforms for lymph node metastasis detection.

Platform	Task	Input	Output	Reference
Paige Breast Lymph Node	Lymph node metastasis detection	H&E-stained sentinel lymph node WSIs	Detection of breast cancer lymph node metastases, including micrometastases and isolated tumor cells (ITCs)	[109]
Aiforia Breast Cancer Suite Lymph Node Metastasis	Lymph node metastasis detection and quantification	H&E-stained lymph node WSIs	Detection and quantification of lymph node metastases from breast, melanoma, and colorectal cancers	[110]
DeepBio SLNB (Sentinel Lymph Node Biopsy)	Sentinel lymph node metastasis detection	Sentinel lymph node H&E WSIs	Detection of metastatic foci, tumor visualization, and automated tumor burden quantification	[145]
Mindpeak Breast Metastasis Detection—Under development	Lymph node metastasis detection	H&E-stained lymph node WSIs	AI tool under development for the detection and visualization of breast cancer metastases in H&E-stained lymph node tissue.	[112]
Primaa Cleo Breast	Lymph node metastasis detection	H&E-stained lymph node WSIs	Detection of macrometastases and heatmap visualization	[131]
Visiopharm Metastasis Detection	Lymph node metastasis detection and quantification	H&E-stained lymph node WSIs	Detection, measurement, and ranking of lymph node metastases in breast and colorectal cancers	[108]
Ibex Breast Ibex Lymph Node- Under development	Lymph node metastasis detection	H&E-stained lymph node WSIs	Detection of lymph node metastases	[111]

**Table 6:** AI platforms for prognostic assessment and risk stratification.

Platform	Task	Input	Output	Reference
Stratipath Breast	Prognostic risk prediction	H&E-stained WSIs	Prognostic risk profiling and patient stratification into low- and high-risk groups for disease progression	[146]
Waiv RlapsRisk BC	Recurrence risk prediction	H&E-stained WSIs	Recurrence risk prediction and patient stratification into low- and high-risk groups	[147]
PreciseDx PreciseBreast	Recurrence risk prediction	H&E-stained WSIs	Recurrence risk prediction and patient stratification into low- and high-risk groups	[148]

**Table 7:** Regulatory classification of AI platforms in breast pathology (verified in July 2026).

Platform	Module	Intended Use	Regulatory Status	Jurisdiction	Reference
Aiforia Technologies Plc, (Helsinki, Finland)	Breast Cancer Grading	Automated breast cancer grading and DCIS detection on H&E WSIs.	CE-IVD	EU	[110]
	ER	Automated ER scoring on breast cancer WSIs.	CE-IVD	EU	
	PR	Automated PR scoring on breast cancer WSIs.	CE-IVD	EU	
	HER2	Automated HER2 scoring on breast cancer WSIs.	CE-IVD	EU	
	Ki-67	Ki-67 proliferation index assessment.	CE-IVD	EU	
	Lymph Node Metastasis	Automated lymph node metastasis detection and quantification on WSIs.	CE-IVD	EU	
Visiopharm A/S (Hørsholm, Denmark)	ER APP	Automated ER-positive and total tumor nuclei quantification with Allred scoring.	CE-IVD	EU	[108]
	PR APP	Automated PR scoring on breast cancer WSIs.	CE-IVD	EU	
	HER2 IHC APP	Automated HER2 IHC scoring on breast cancer WSIs.	CE-IVD	EU	
	Ki-67 APP	Automated Ki-67 proliferation index assessment on breast cancer WSIs.	CE-IVD	EU	
	Metastasis Detection APP	Automated detection and quantification of lymph node metastases on H&E WSIs.	CE-IVD	EU	
	Invasive Tumor Detection	Automated detection of invasive tumor regions for biomarker analysis on breast cancer WSIs.	CE-IVD	EU	
Roche Diagnostics International Ltd. (Rotkreuz, Switzerland)	uPath ER (SP1)	Automated ER scoring within pathologist-annotated invasive tumor regions.	RUO	International/ Research use	[113]
	uPath PR (1E2)	Automated PR scoring within pathologist-annotated invasive tumor regions.	RUO	International/ Research use	
	uPath Ki-67 (30-9)	Automated Ki-67 scoring within pathologist-annotated viable tumor regions.	RUO	International/ Research use	
	uPath HER2 (4B5) by navify^®^ Digital Pathology	Automated HER2 (0–3+) scoring within pathologist-annotated viable tumor regions.	RUO	International/ Research use	
	uPath HER2 Dual ISH	Automated HER2/Chr17 ratio assessment within pathologist-annotated viable tumor regions.	RUO	International/ Research use	
Ibex Breast Medical Analytics Ltd. (Tel Aviv, Israel)	Ibex Breast H&E	Automated detection and classification of benign and malignant breast lesions on H&E WSIs.	CE-IVD RUO (US)	EU US	[111]
	IBEX ER/PR	Automated ER/PR quantification in invasive breast carcinoma with automated DCIS exclusion.	CE-IVD RUO (US)	EU US	
	IBEX HER2	Automated HER2 IHC scoring, including HER2-low and HER2-ultralow assessment.	CE-IVD RUO (US)	EU US	
	IBEX Ki-67	Automated Ki-67 quantification and proliferation index assessment on breast WSIs.	CE-IVD RUO (US)	EU	
Mindpeak Breast GmbH (Hamburg, Germany)	H&E Breast	Automated tissue segmentation and invasive tumor detection on routine H&E slides.	RUO	International/ Research use	[112]
	ER Breast	Automated ER expression quantification	CE-IVD	EU	
	PR	Automated PR expression quantification.	CE-IVD	EU	
	HER2	Automated HER2 status assessment, including HER2-low and HER2-ultralow expression.	RUO (standard version); CE-IVD (RoI variant, EU)	International/ Research use EU	
	Ki-67	Automated quantification of the Ki-67 proliferation index using whole-slide analysis.	CE-IVD	EU	
	PD-L1 Breast	Automated PD-L1 expression assessment using Combined Positive Score (CPS).	RUO	International/ Research use	
	p53	Automated quantification of p53 expression.	RUO	International/ Research use	
Paige AI, Inc. (New York, NY, USA)	Paige Breast Detect	Detection and localization of suspicious breast cancer and pre-neoplastic lesions on H&E WSIs.	CE-IVDD	EU	[109]
	Paige Breast Mitosis	Automated mitotic figure detection, hotspot identification, and mitotic count/density assessment on H&E WSIs.	CE-IVDD	EU	
	Paige Breast Lymph Node	Detection of suspicious breast cancer metastases in lymph node WSIs.	CE-IVDD	EU	
	HER2 Complete	AI-based prediction of HER2 expression from H&E WSIs, including identification of HER2-low tumors.	CE-IVDD	EU	
PathAI, Inc. (Boston, MA, USA)	TumorDetect	AI-assisted case prioritization for suspected malignant solid tumors on H&E WSIs.	RUO	International/ Research use	[129]
	AIM-TumorCellularity	Automated quantification of tumor cellularity on H&E whole-slide images to support NGS tissue assessment.	RUO	International/ Research use	
	AIM-ER Breast	Automated quantification of ER expression in invasive breast carcinoma.	RUO	International/ Research use	
	AIM-PR Breast	Automated quantification of PR expression in invasive breast carcinoma.	RUO	International/ Research use	
	AIM-HER2 Breast	Automated HER2 scoring in invasive breast carcinoma.	RUO	International/ Research use	
	AIM-Ki67 Breast	Automated quantification of Ki-67 expression in invasive breast carcinoma.	RUO	International/ Research use	
Indica Labs, Inc. (Albuquerque, NM, USA) [142]	HALO Macrodissection AI	AI-assisted tumor content quantification and ROI selection for macrodissection of H&E breast cancer specimens.	RUO	International/ Research use	[134]
	Breast IHC AI	Automated AI-based scoring of HER2 (including low/ultralow), ER, PR, and Ki-67 on breast IHC WSIs.	RUO	International/ Research use	[141]
DeepBio Inc. (Seoul, Republic of Korea) [130]	DeepDx Breast—Resection	AI-assisted detection of IDC/DCIS regions and tumor-to-tissue ratio estimation on breast resection.	RUO	International/ Research use	[140]
	DeepDx Breast—SLNB	AI-assisted detection and visualization of breast cancer metastases in sentinel lymph node WSIs, with automatic tumor ratio quantification.	RUO	International/ Research use	[145]
Primaa SAS (Paris, France)	Cleo Breast (H&E)	AI-powered all-in-one primary diagnostic solution for breast cancer on WSIs, providing automated detection and quantification of breast tissue biomarkers.	CE-IVDR	EU	[131]
Gestalt Diagnostics, Inc. (Spokane, WA, USA)	Mitotic Figure Detection	AI-assisted detection of mitotic figures and automatic identification of the 10 HPF region with the highest mitotic activity on H&E WSIs.	RUO	International/ Research use	[133]
Waiv/Owkin Dx (Paris, France)	RlapsRisk BC	AI-based prognostic risk assessment for early ER+/HER2− breast cancer from routine H&E WSIs.	CE-IVDR	EU	[147]
	BRCAura	AI-based prediction of germline BRCA1/2 mutation status from H&E WSIs of HR+/HER2− breast cancer.	RUO	International/ Research use	[135]
Stratipath AB (Stockholm, Sweden)	Stratipath Breast	AI-based prognostic risk stratification of invasive breast cancer from routine H&E WSIs.	CE-IVD	EU	[146]
Lunit Inc. (Seoul, Republic of Korea)	SCOPE IO	AI-powered immune phenotyping and tumor microenvironment (TME) feature analysis from H&E WSIs.	RUO	International/ Research use	[136]

## Data Availability

No new data were created or analyzed in this study. Data sharing is not applicable to this article.

## Abbreviations

The following abbreviations are used in this manuscript:

AcCC	Acinic Cell Carcinoma
AdDC	Adenoid Cystic Carcinoma
AI	Artificial Intelligence
AJCC	American Joint Committee on Cancer
ALH	Atypical Lobular Hyperplasia
AR	Androgen Receptor
AUC	Area Under the Curve
BC	Breast cancer
BLBC	Basal-Like Breast Cancer
BRCA	Breast Cancer Gene (BRCA1/2)
CAM	Class Activation Mapping
CE-IVD	Conformité Européenne—In Vitro Diagnostic
CE-IVDD	Conformité Européenne—In Vitro Diagnostic Medical Devices Directive
CE-IVDR	Conformité Européenne—In Vitro Diagnostic Regulation
CIS	Carcinoma in situ
CK	Cytokeratin
CNN	Convolutional Neural Network
CPS	Combined Positive Score
DCIS	Ductal Carcinoma in situ
DL	Deep Learning
DP	Digital Pathology
ER	Estrogen Receptor
FDA	Food and Drug Administration
Grad-CAM	Gradient-weighted Class Activation Mapping
H&E	Hematoxylin and Eosin
HER2	Human Epidermal Growth Factor Receptor 2
HPF	High-Power Field
HR	Hazard Ratio
IDC	Invasive Ductal Carcinoma
IBC-NST	Invasive Breast Carcinoma of No Special Type
ICC	Invasive Cribriform Carcinoma
IHC	Immunohistochemistry
ILC	Invasive Lobular Carcinoma
IMPC	Invasive Micropapillary Carcinoma
IPC	Invasive Papillary Carcinoma
Ki-67	Proliferation Index Marker
LCIS	Lobular Carcinoma in Situ
LVI	Lymphovascular Invasion
MEC	Mucoepidermoid Carcinoma
MIL	Multiple Instance Learning
ML	Machine Learning
NEC	Neuroendocrine Carcinoma
NET	Neuroendocrine Tumor
NGS	Next-Generation Sequencing
PAC	Polymorphous Adenocarcinoma
PAS-D	Periodic Acid–Schiff with Diastase
PAM50	Prediction Analysis of Microarray 50
PD-L1	Programmed Death-Ligand 1
PR	Progesterone Receptor
ROI	Region of Interest
RUO	Research Use Only
SBC	Secretory Breast Carcinoma
SLNB	Sentinel Lymph Node Biopsy
SMA	Smooth Muscle Actin
SVM	Support Vector Machine
TC	Tubular Carcinoma
TCGA	The Cancer Genome Atlas
TCCRP	Tall Cell Carcinoma with Reversed Polarity
TILs	Tumor-Infiltrating Lymphocytes
TME	Tumor Microenvironment
TNM	Tumor–Node–Metastasis Classification
UKCA	UK Conformity Assessed
ViT	Vision Transformer
WHO	World Health Organization
WSI	Whole-Slide Image
XAI	Explainable Artificial Intelligence
